# Scaffold-Based Pan-Agonist Design for the PPARα, PPARβ and PPARγ Receptors

**DOI:** 10.1371/journal.pone.0048453

**Published:** 2012-10-31

**Authors:** Li-Song Zhang, Shu-Qing Wang, Wei-Ren Xu, Run-Ling Wang, Jing-Fang Wang

**Affiliations:** 1 Tianjin Key Laboratory on Technologies Enabling Development of Clinical Therapeutics and Diagnostics (Theranostics), School of Pharmacy, Tianjin Medical University, Tianjin, China; 2 Tianjin Institute of Pharmaceutical Research (TIPR), Tianjin, China; 3 Key Laboratory of Systems Biomedicine (Ministry of Education), Shanghai Center for Systems Biomedicine, Shanghai Jiao Tong University, Shanghai, China; 4 Shanghai Center for Bioinformation Technology, Shanghai, China; Semmelweis University, Hungary

## Abstract

As important members of nuclear receptor superfamily, Peroxisome proliferator-activated receptors (PPAR) play essential roles in regulating cellular differentiation, development, metabolism, and tumorigenesis of higher organisms. The PPAR receptors have 3 identified subtypes: PPARα, PPARβ and PPARγ, all of which have been treated as attractive targets for developing drugs to treat type 2 diabetes. Due to the undesirable side-effects, many PPAR agonists including PPARα/γ and PPARβ/γ dual agonists are stopped by US FDA in the clinical trials. An alternative strategy is to design novel pan-agonist that can simultaneously activate PPARα, PPARβ and PPARγ. Under such an idea, in the current study we adopted the core hopping algorithm and glide docking procedure to generate 7 novel compounds based on a typical PPAR pan-agonist LY465608. It was observed by the docking procedures and molecular dynamics simulations that the compounds generated by the core hopping and glide docking not only possessed the similar functions as the original LY465608 compound to activate PPARα, PPARβ and PPARγ receptors, but also had more favorable conformation for binding to the PPAR receptors. The additional absorption, distribution, metabolism and excretion (ADME) predictions showed that the 7 compounds (especially Cpd#1) hold high potential to be novel lead compounds for the PPAR pan-agonist. Our findings can provide a new strategy or useful insights for designing the effective pan-agonists against the type 2 diabetes.

## Introduction

Type 2 diabetes are characterized by hyperglycemia, insulin resistance and defects in insulin secretion, thus the patient with type 2 diabetes often suffer from symptoms of dyslipidemia, hypertension as well as obesity [1]. As reported, there are nearly 151 million individuals in the world affected type 2 diabetes, which will increase to 346 million at the end of 2012 [2]. As important members of nuclear receptor superfamily, Peroxisome proliferator-activated receptors (PPAR) play essential roles in regulating cellular differentiation, development, metabolism, and tumorigenesis of higher organisms [3], thus these receptors have been considered as attractive targets for treating type 2 diabetes. PPAR receptors have 3 identified subtypes: PPARα, PPARβ and PPARγ [4]. Although the ligand-binding domains of the subtypes share 60%–70% sequence similarities, they have specific organization distributions and active functions.

PPARα is expressed at high level in the brown adipose tissue, liver, kidney, heart, and skeletal muscle [5]. This PPAR subtype has significant functions in fatty acid metabolism and energy homeostasis [6] as well as modification of the high-density lipoprotein (HDL) circulations [7]. The activation of PPARα receptor is propitious to improve glucose tolerance so as to decrease the risks of developing atherosclerotic lesions [8]. Due to its crucial role in the fatty acid oxidation in the liver cells, PPARα can increase the energy consumption so as to achieve the controlling of energy metabolism and body weight [9]. Thus, this PPAR receptor can be used as an anti-obesity target, against which a number of clinical drugs (i.e., beclofibrate, bezafibrate, ciprofibrate, clofibrate, fenofibrate and gemfibrozil) have been approved by US Food and Drug Administration (FDA) to treat the dyslipidemia.

PPARβ is widely distributed in various body tissues with responsibility for controlling blood lipid concentration and insulin sensitivity [10]. Experimental evidences show that this PPAR receptor functions as a regulator in fatty acid catabolism, energy balance and cholesterol reversion transportation [11]. In animal models of type 2 diabetes, this receptor is found to have the ability of improving insulin resistance and decreasing plasma glucose [12], [13]. PPARγ is expressed in adipose tissue, macrophages and vascular smooth muscles at high levels. The activation of this PPAR receptor can increase the differentiation of the fat cells, improve the storage of fatty acids and enhance insulin sensitivity in the adipose tissue, skeletal muscle and liver [6], [14].

As attractive targets for type 2 diabetes, many PPAR agonists, including PPARα/γ and PPARβ/γ dual agonists, have been designed and synthesized. Due to the undesirable side-effects, many PPAR agonists (i.e., muraglitazar, tesaglitazar, ragaglitazar, TAK559 and KRP297) are stopped by US FDA in the clinical trials [15], [16], [17], [18]. An alternative strategy is to design novel agonist that can simultaneously activate PPARα, PPARβ and PPARγ with an aim to treat both insulin resistance and dyslipidemia [19]. In the current study, we employed molecular modeling technologies with a core hopping approach to screen the fragment database, with an aim of searching for novel PPAR pan-agonists to treat type 2 diabetes.

## Materials and Computational Methods

### 1. Initial Structures for PPARα, PPARβ and PPARγ Receptors

The initial structures of PPARα, PPARβ and PPARγ receptors were obtained from the Protein Data Bank (PDB) with PDB entries of 1k7l.pdb [20], 1gwx.pdb [6] and 1k74.pdb [20], respectively. The crystal structure of PPARβ was released in 2000 with a resolution of 2.5 Å by Xu et al [6], who released the crystal structures of PPARα and PPARγ one year later [20], with resolutions of 2.5 Å and 2.3 Å, respectively. Except for the polar hydrogen and heavy atoms, all the other atoms including non-polar hydrogen atoms in these crystal structures were removed. Hydrogen atoms were subsequently added to the receptor structures of all the PPAR receptors based on the computational pKa values for each residue in the crystal structures. Finally the structural models for PPARα, PPARβ and PPARγ receptors were obtained after a steepest descent energy minimization with OPLS force field parameters [21].

### 2. Molecular Docking Procedure with a Core Hopping Approach

Molecular docking technology has been increasingly used in the course of drug research and development [22], [23], [24]. To improve the activity of a lead compound, we usually vary the side chains attached to a core part of the compound, because in many cases it is the side chains that bind to the receptors [25]. However, it also makes sense to vary the core structure to find novel compounds (scaffolds). In the present study, core hopping algorithm (CombiGlid 2.5. Schrodinger LLC, New York, 2009) was adopted during the molecular docking procedure with the functions to perform both fragment-based replacing and docking. The core hopping strategy is to screen multiple scaffolds against a guiding structure, searching for alignments of potential attachment points on the scaffold with the attachment points on the guiding structure. Generally speaking, the core hopping process contains 4 major steps:

The first step is to define some possible points at which the cores are attached to the scaffold. It is also the define combinations step from the Combinatorial Screening panel packaged in Schrodinger (www.schrodinger.com). The second step is to define the receptor grid file in the Receptor Preparation panel in Schrodinger. The third step is the core preparation with the Protocore Preparation module in Schrodinger to find the core attaching to the scaffold using the fragment database derived from ZINC database (http://zinc.docking.org). The last step is to align and dock the entire molecular structures built up by the core and scaffold into PPAR receptors. The cores are sorted and filtered by goodness of alignment and then redocked into the receptor after attaching the scaffold, followed by using the docking scores to sort the final molecules.

### 3. Molecular Dynamics Simulation

The candidates obtained from the core hopping docking procedure were then subjected to a series of molecular dynamic simulations by the open software GROMACS 4.0 [26]. The PPAR receptors were parameterized by GROMOS 96–53a6 force field parameters [27], while the topology file, partial charges and force field parameters of the candidates were generated by the online tool PRODRG [28]. The simulation systems were subsequently inserted into 108 DPPC lipid bilayers, and solvated in a specific box with SPC water and a space of 9 Å around the solute. To neutralize the redundant charges of the simulation systems, 4 sodium ions were added into the simulation systems to randomly replace 4 water molecules. The neutralized systems were subjected to an energy minimization for about 3000 steps with the steepest descents approach. Finally, 10-ns molecular dynamic simulations were performed with constant temperature (300K), periodic boundary conditions and NVT ensembles.

During the molecular dynamics simulations, all bonds in the simulation systems were constrained by the Linear Constraint Solver (LINCS) algorithm [29], [30], and the atom velocities for the start-up runs were obtained based on the Maxwell distribution at 300 K [31], [32]. To maintain the simulation systems at a constant temperature and volume, the Berendsen thermostat with a coupling time of 0.1 ps and v-rescale scheme were applied [33]. The electrostatic interactions were treated by the particle mesh Ewald (PME) algorithm with interpolation order of 4 and a grid spacing of 0.12 nm [34], [35]. The van der Waals interactions were treated by using a cutoff of 12 Å [36], [37]. The integration step was set to 2 fs, and the coordinates were saved every 1 ps.

### 4. ADME Prediction

In the current study, QikProp was employed for predicting the absorption, distribution, metabolism and excretion (ADME) properties for all the candidates. QikProp is designed by Prof. William L. Jorgensen, and can predict physically significant descriptors (i.e., the partition coefficient, van der Waals surface area of polar nitrogen and oxygen atoms, and predicted aqueous solubility) and pharmaceutically relevant properties for small drug-like molecules. This software also provides ranges for comparing a particular properties of molecules with those of 95% known drugs, and flags 30 types of reactive functional groups that may cause false positives in high-throughput screening assays.

## Results and Discussion

### 1. ligand-binding Domains of the PPAR Receptors

**Figure 1:**
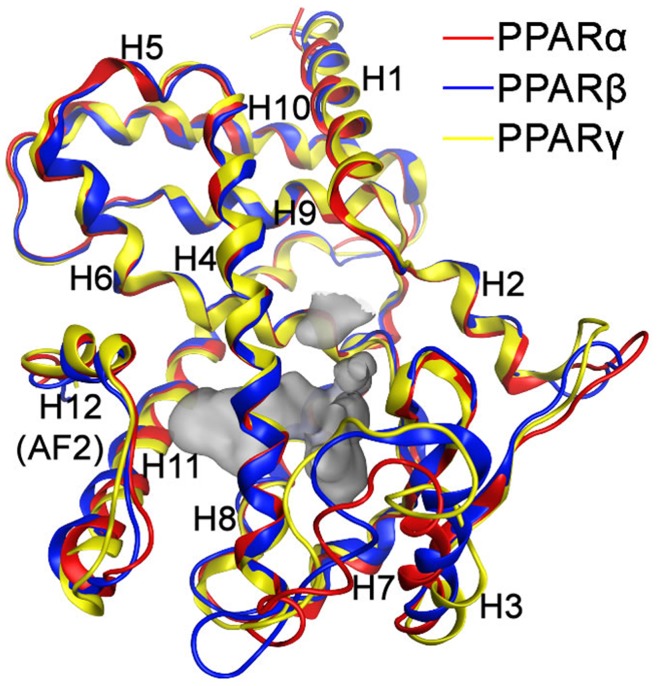
The ligand-binding domains of PPARα, PPARβ and PPARγ receptors. The ligand-binding domains for PPARα (red), PPARβ (blue) and PPARγ (yellow) receptors share some common features: 1) composed of 12 α-helices arranged in an antiparallel helix sandwich, and a 4-stranded antiparallel β sheet; 2) Y-shaped hydrophobic ligand binding pocket with a volume of ∼1300 cubic angstroms; and 3) a C-terminal helix (Helix 12 or AF2 helix) showing widely conformational variations in different crystals and playing essential roles in activation of PPAR receptors.

Experimental evidences showed that the biological functions of PPAR receptors is regulated by the precise shape of their ligand-binding domain induced by the binding of ligands including a number of coactivators and corepressor proteins. These ligands can significantly simulate or inhibit PPAR receptor functions. According to the X-ray crystallography studies, the ligand-binding domains of PPARα, PPARβ and PPARγ receptors are very similar with the RMS deviations between Cα atoms less than 1 Å. Besides, they also share some common features (**Figure 1**): i) composed of 12 α-helices arranged in an antiparallel helix sandwich, and a 4-stranded antiparallel β sheet; ii) Y-shaped hydrophobic ligand binding pocket with a volume of ∼ 1300 cubic angstroms; and iii) a C-terminal helix (Helix 12 or AF2 helix) showing widely conformational variations in different crystals and playing essential roles in activation of PPAR receptors.

Addition to the common features mentioned above, the ligand-binding domains for PPARα, PPARβ and PPARγ receptors also have their unique features. For instance, it is significantly narrower in the region adjacent to the AF2 helix in PPARβ, which is not suitable for the ligands containing larger polar heads. Compared with the PPARβ ligand-binding domain, the one of PPARα is comparatively more lipophilic, while the one of PPARγ is more hydrophilic. The largest variation among the three ligand-binding domains is found in the omega loop displaying a comparatively high RMS deviation and differing among a number of reported crystal structures.

### 2. Core Hopping and Drug Design

**Figure 2:**
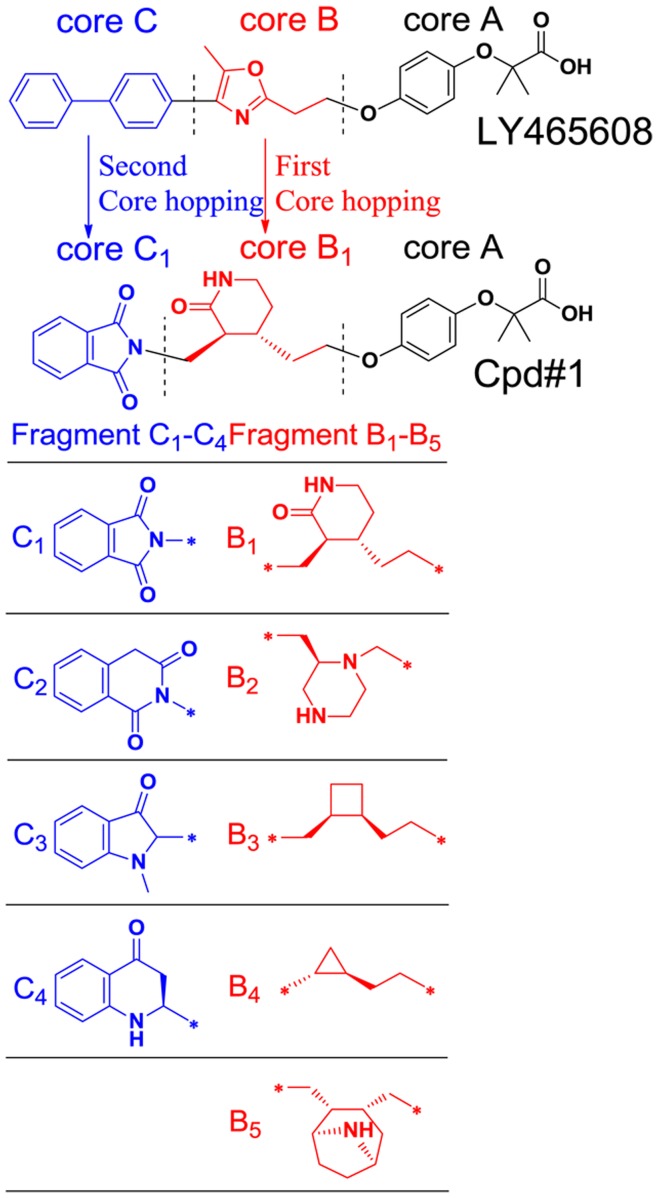
Diagrammatically showing the core hopping procedure. The Original LY465608 structure contains 3 major components: 3 major components: a polar acidic head (Core A), a linker group (Core B) and a hydrophobic tial (Core C). Owe to forming significant hydrogen bonds with the ligand-binding domain, the Core A would be retained during the core hopping procedure. The 1^st^ core hopping operation was aimed at the Core B to generate 5 scaffolds named Fragment B_1_ to B_5_ respectively. The 2^nd^ core hopping operation was aimed at the Core C to generate 4 scaffolds, named Fragment C_1_ to C_4_, respectively. Thus, a total of 20 combinations were obtained.

During the core hoping processes, LY465608 is selected as a guiding structure. This small molecule is firstly reported as a PPARα/γ dual agonist, latter proved to excite PPARβ as well. Owe to playing a positive role in keeping lipid and cholesterol homeostasis stability by reducing serum triglycerides and increasing HDL cholesterol, LY465608 has been treated as a typical PPAR pan-agonist to lower plasma glucose levels and improve insulin sensitivity. The structure of LY465608 can be divided into 3 major components (**Figure 2**): a polar acidic head (Core A), a linker group (Core B) and a hydrophobic tial (Core C). The Core A contains an acidic head, and is reported to be of most importance for the ligand-binding and PPAR receptor activation. Thus, this part will be retained during the entire core hopping processes as described below.

The 1^st^ core hoppling operation was aimed at the Core B (see the red part of **Figure 2**) to generate 5 scaffolds, named Fragment B_1_ to B_5_ respectively. The 2^nd^ core hopping operation was aimed at the Core C (see the blue part of **Figure 2**) to generate 4 scaffolds, named Fragment C_1_ to C_4_, respectively. Consequently, a total of 20 combinations of LY465608 derivatives were thus obtained. Subsequently, each of the 20 derivatives was docked into PPARα (1k7l.pdb), PPARβ (1gwx.pdb) and PPARγ receptors (1k74.pdb), respectively. The 20 derivative compounds were then ranked roughly according to their docking scores to PPARα, PPARβ and PPARγ receptors, respectively. The derivative compounds that employed stronger binding affinities than the original LY465608 with all the PPAR receptors were listed in **Table 1** (the chemical structures of the derivative compounds mentioned above were included in **Table S1**). Of the top 7 derivative compounds, the Cpd#1 with Core A-Fragment B_1_-Fragment C_1_ has the strongest binding affinity with all the PPAR receptors, and hence it was singled out for further studies.

**Table 1:** The top 7 hits in the core hopping and glide docking. For comparison, the typical PPAR pan-agonists bezafibrate, LY465608 and GW677954 are also involved.

Compound	Docking score			Key residues		
	PPARα	PPARβ	PPARγ	PPARα	PPARβ	PPARγ
Bezafibrate	−10.53	−10.17	−11.82	Y464,H440Y314,S280	Y473,H449H323	Y473,Y449H323,S289
LY465608	−10.28	−12.62	−10.64	Y464,H440Y314,S280	Y473,H449H323	Y473,Y449H323,S289
GW677954	−12.49	−13.50	−8.74	Y464,H440Y314,S280	Y473,H449H323	Y473,Y449H323
Cpd#1	−12.54	−13.00	−13.01	Y464,H440Y314,S280	Y473,H449H323,T288	Y473,Y449H323,S289
Cpd#2	−11.40	−12.40	−12.58	Y464,Y314S280	Y473,H449H323,T288	Y473,Y449H323,S289
Cpd#3	−12.40	−13.40	−12.39	Y464,H440Y314,S280	Y473,H449H323,T288	Y473,Y449H323,S289
Cpd#4	−12.00	−13.51	−13.49	Y464,H440Y314,A333	Y473,H449H323,T288	Y473,Y449H323,S289
Cpd#5	−10.95	−11.56	−11.88	Y464,H440Y314,S280	Y473,H449H323,T288	Y473,Y449H323,S289
Cpd#6	−11.64	−10.93	−12.99	Y464,H440Y314,S280	Y473,H449H323	Y473,Y449H323,S289
Cpd#7	−12.09	−12.49	−13.11	Y464,H440Y314,S280	Y473,H449H323	Y473,Y449H323,S289

**Figure 3:**
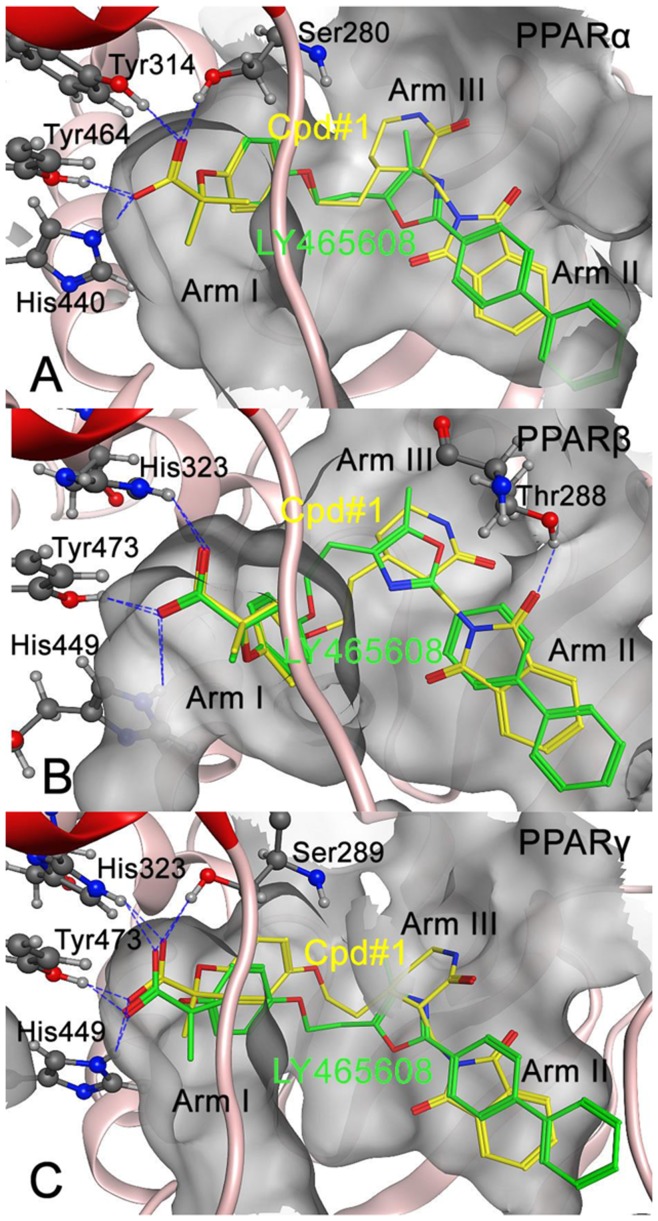
Diagrammatically showing the favorable conformation obtained by docking Ly465608 and Cpd#1 into (A) PPARα, (B) PPARβ and (C) PPARγ, respectively. The binding pocket in the current study is defined by those residues with its heavy atoms within a distance limitation of 5 Å from LY465608 or Cpd#1. The AF2 function domain is shown in red helix, and the hydrophobic surfaces of the ligand-binding domain are colored in green. The dotted lines show the hydrogen bonding interactions between the receptors and ligands.

As shown in **Figure 3**, the favorable binding mode of the Cpd#1 was aligned with the guiding structure LY465608 for PPARα, PPARβ and PPARγ receptors. According to the crystal studies, a conversed hydrogen bonding network composed of 4 significant hydrogen bonds formed by the acidic head of both LY465608 and Cpd#1 to the active site residues (Ser280, Tyr314, Tyr464 and His440 in PPARα receptor; Ser289, His323, Tyr473 and His449 in PPARγ receptor) of PPARα and PPARγ receptors were observed in our docking results. However, in PPARβ receptor 3 hydrogen bonds were detected between the acidic head of both LY465608 and Cpd#1 and the active site residues (His323, His449and Tyr473). Based on the previous experimental and theoretical studies, these hydrogen bonds were believed to play crucial roles in stabilizing the AF2 helix in the active conformation, which is essential for the ligand-binding and receptor activation.

Addition to the conserved hydrogen bonding network mentioned above, Cpd#1 formed an additional hydrogen bond with Thr288 in PPARβ receptor, which could notably enhance the binding affinity compared to the guiding molecule LY465608. The hydrophobic tail of the Core C in both LY465608 and Cpd#1 were buried well in the hydrophobic pocket of the ligand-binding domains of PPARα, PPARβ and PPARγ receptors. However, due to the suitable size, Cpd#1 was more fitted to the hydrophobic arm II, resulting in the stronger binding affinities than LY465608 (also see **Table 1**).

### 3. Dynamics Behaviors of the Cpd#1-PPAR Receptors

**Figure 4:**
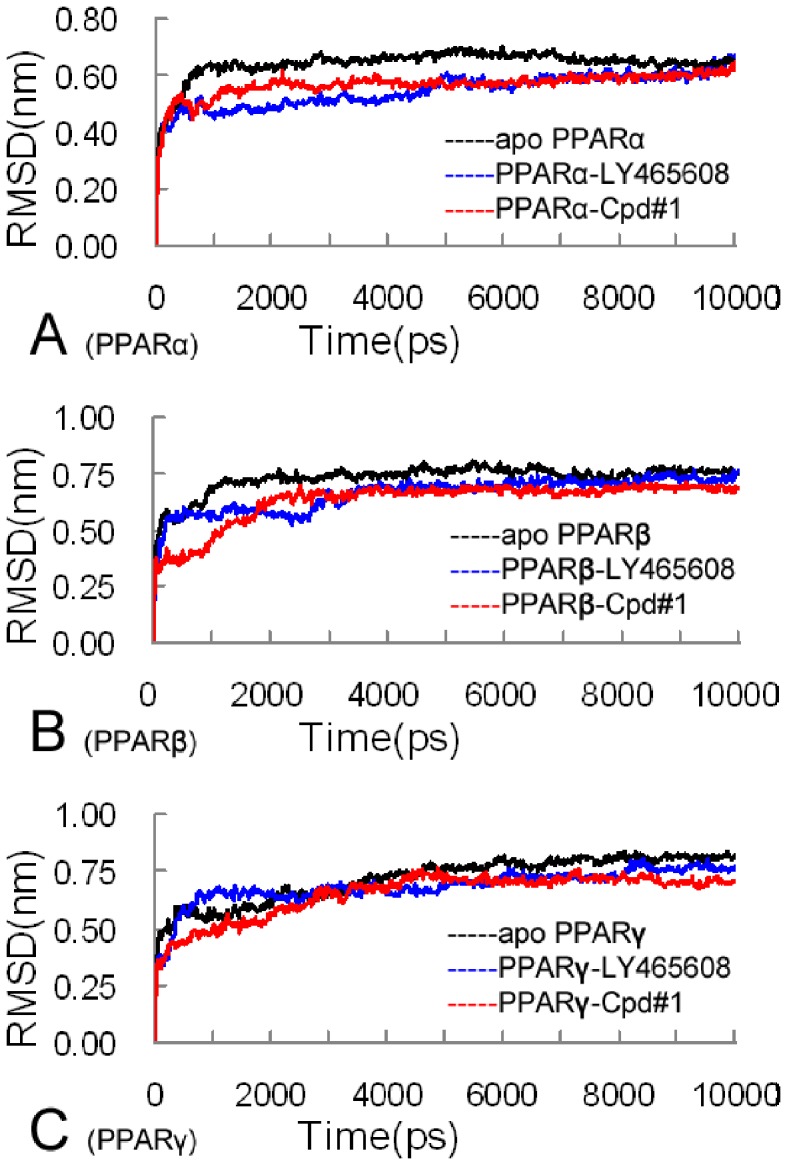
The RMS deviations for the backbone structures of the apo, LY465608-bound and Cpd#1-bound states of PPARα, PPARβ and PPARγ receptors. Both the fluctuations of total RMS deviations and final RMS deviations for all the simulations systems are no more than 1 Å during our molecular dynamics simulations, indicating that all the simulation systems are in the equilibrium states.

Molecular dynamics can provide useful information for characterizing the internal motions of biomacromolecules with time [38], [39], [40]. To study the dynamics behaviors of the Cpd#1-PPAR receptors, 10-ns molecular dynamics simulations were performed on apo, LY465608-bound and Cpd#1-bound states of PPARα, PPARβ and PPARγ receptors. The root mean square (RMS) deviation from the initial structure is considered as an important criterion usually used to measure the convergence of the protein systems concerned. In the current case, the final RMS deviation values of the backbone structures for all the simulation systems were no more than 0.8 nm (**Figure 4**), giving an indication that the receptor structures had reached to the equilibrium states with little alterations during the entire simulations.

To further study the conversed hydrogen bonding network mentioned above, 200 snapshots for the LY465608-bound and Cpd#1-bound states of PPARα, PPARβ and PPARγ receptors were retrieved from the last 1-ns segment on the molecular dynamics simulation trajectories with an interval of 5 ps, and statistical analyses were further performed on the conversed hydrogen bonding network (for detailed information, please see **Table 2** and **Table 3**). For the PPARα receptor, the hydrogen bonds formed by the key residues (Ser280, Tyr314, Tyr464 and His440) and both ligands (LY465608 and Cpd#1) were quite similar, no matter the average distances between the heavy atoms of the hydrogen donor and receptor and the hydrogen bond life-time. However, huge differences were detected for PPARβ and PPARγ receptors. For the PPARβ receptor, although the differences between the hydrogen bonds formed by LY465608 and Cpd#1 were quite small, Cpd#1 could form an additional hydrogen bond with Thr288 in the Arm III region. For the PPARγ receptor, the hydrogen bonds formed by Ser289 with LY465608 and Cpd#1 were quite different. Compared with the LY465608-bound system, the hydrogen bond formed by Ser289 in the Cpd#1-bound system employed a much smaller average distance between the heavy atoms and a much larger life-time. This observation indicated that the hydrogen bond formed by Ser289 and Cpd#1 was stronger than that of the LY465608-bound system.

**Table 2:** Detailed information for the conserved hydrogen bonding network formed by LY465608 and key residues in the active site of the PPAR receptors.

Receptors	H Donor	H Receptor	Distance (Å)	Life-time (%)
PPARα	Ser280	LY465608	1.82	32.8
	Tyr314	LY465608	1.76	45.7
	LY465608	His440	1.86	34.6
	LY465608	Tyr464	1.66	54.6
PPARβ	LY465608	His323	2.05	36.6
	His449	LY465608	2.37	15.7
	Tyr473	LY465608	1.80	29.9
PPARγ	LY465608	Ser289	2.10	29.8
	LY465608	His323	1.96	35.9
	His449	LY465608	1.88	38.5
	Tyr473	LY465608	1.74	49.1

**Table 3:** Detailed information for the conserved hydrogen bonding network formed by Cpd#1 and key residues in the active site of the PPAR receptors.

Receptors	H Donor	H Receptor	Distance (Å)	Life-Time (%)
PPARα	Ser280	Cpd#1	1.82	32.6
	Tyr314	Cpd#1	1.76	45.3
	Cpd#1	His440	1.87	34.9
	Cpd#1	Tyr464	1.64	54.7
PPARβ	Cpd#1	His323	1.94	37.1
	His449	Cpd#1	2.38	15.4
	Tyr473	Cpd#1	1.81	30.3
	Thr288	Cpd#1	1.95	36.2
PPARγ	Cpd#1	Ser289	1.68	71.9
	Cpd#1	His323	1.87	36.4
	His449	Cpd#1	1.88	38.3
	Tyr473	Cpd#1	1.64	68.7

### 4. AMDE Predictions

Absorption, distribution, metabolism and excretion (ADME) describes the druggable dispositions of the lead compounds in pharmacokinetics and pharmacology. In our study, some pharmaceutically relevant properties of the new designed agonist derivatives as well as the original LY465608 compound, such as the “molecular mass” (Mol_MW), “hydrogen bond donors” (DonorHB), “hydrogen bond acceptor” (AccptHB), “partition coefficient” (logP o/w), “van der Waals surface area of polar nitrogen and oxygen atoms” (PSA), and “aqueous solubility” (logS), were predicted by means of the QikProp module packaged in the Schrodinger 2009. The predicted ADME results were listed in **Table 4**.

**Table 4:** The ADME predictions for the top 7 hits in the core hopping and glide docking.

Compound	Mol_MW^a^	DonorHB^b^	AccptHB^c^	logP o/w^d^	PSA^e^	logS^f^
Bezafibrate	361.82	2.00	5.25	4.37	81.5	−5.88
LY465608	457.53	1.00	5.50	6.69	82.0	−8.13
GW677954	499.54	1.00	4.75	6.91	63.4	−7.94
Cpd#1	480.52	2.00	9.00	3.02	157.5	−4.30
Cpd#2	453.49	2.00	10.0	0.23	134.3	−3.10
Cpd#3	451.52	1.00	6.50	5.06	108.2	−6.02
Cpd#4	480.56	2.00	9.00	1.22	130.4	−4.29
Cpd#5	478.54	2.00	8.00	1.99	129.5	−5.63
Cpd#6	409.44	1.00	6.50	4.27	118.0	−5.38
Cpd#7	423.58	1.00	6.50	4.86	94.0	−6.31

a The molecular mass (Mol_MW) should be less than 500 Daltons.

b The hydrogen bond donors (Donor HB) should be not more than 5.

c The hydrogen bond acceptors (AccptHB) should be not more than 10.

d The predicted octanol/water partition coefficient (−0.4 to 5.6).

e The van der Waals surface area of polar nitrogen and oxygen atoms (7.0 to 200.0).

f The predicted aqueous solubility S (mol dm^–3^) is the concentration of the solute in a saturated solution that is in equilibrium with the crystalline solid (−6.5 to 0.5).

For comparison, the typical PPAR pan-agonists bezafibrate, Ly465608 and GW677954 are also involved.

According to the Lipinski’s Rule of Five, the orally active drugs should have no more than one violation of the following criteria: i) no more than 5 hydrogen bond donors (DonorHB ≤5); ii) no more than 10 hydrogen bond acceptors (AccptHB ≤10); iii) molecular mass less than 500 Daltons (Mol_MW ≤500); and iv) the octanol-water partition coefficient logP in −0.4 to 5.6 range (−0.4≥ logP o/w ≤5.6). All the criteria for the original LY465608 compound were within the acceptable ranges of the Lipinski’s Rule of Five, except the logP o/w value which was comparatively higher and had high-risk to induce unfavorable distributions of the compound on fat and body fluid. This also might be the reason why the original LY465608 compound shows comparatively high toxicity. However, after rational design with the core hopping approach, the ADME properties of the 7 candidates are all within the acceptable range for human beings, indicating that these derivatives found in the current study can be utilized as potential candidates for the purpose of developing novel drugs.

In summary, we employed the core hopping approach to generate a series of novel compounds with an aim of finding new and powerful pan-agonists for PPARα, PPARβ and PPARγ receptors. After flexible docking procedures and molecular dynamics simulations, a set of 7 novel compounds were found using LY465608, a typical PPAR pan-agonist, as a guiding structure. Compared with the existing pan-agonist LY465608, the 7 candidates found in the core hopping processes not only had the similar function in activating PPARα, PPARβ and PPARγ receptors, but also assumed the conformation more favorable in binding to the PPAR receptors. It is anticipated that the 7 candidates, especially Cpd#1, may become potential lead compounds for designing novel pan-agonist against PPARα, PPARβ and PPARγ receptors with comparatively lower toxicities.

## Supporting Information

Table S1 **The chemical structures of the top 7 hits in the core hopping and glide docking.** For comparison, the typical PPAR pan-agonists bezafibrate, Ly465608 and GW677954 are also involved.
(DOC)
