# Supplementary material for: Scaffold-Based Pan-Agonist Design for the PPARα, PPARβ and PPARγ Receptors
Source: PLoS One. 2012 Oct 31;7(10):e48453. doi: 10.1371/journal.pone.0048453 (PMC3485212; doi:10.1371/journal.pone.0048453)
Supplement: Table S1 — The chemical structures of the top 7 hits in the core hopping and glide docking. For comparison, the typical PPAR pan-agonists bezafibrate, Ly465608 and GW677954 are also involved. (DOC) [file pone.0048453.s001.doc]

**Table S1.** The chemical structures of the top 7 hits in the core hopping and glide docking. For comparison, the typical PPAR pan-agonists bezafibrate, Ly465608 and GW677954 are also involved.

| Compound | Docking score | | | Key residues | | |
| --- | --- | --- | --- | --- | --- | --- |
|  | PPARα | PPARβ | PPARγ | PPARα | PPARβ | PPARγ |
|  | -10.53 | -10.17 | -11.82 | Y464,H440  Y314,S280 | Y473,H449  H323 | Y473,Y449  H323,S289 |
|  | -10.28 | -12.62 | -10.64 | Y464,H440  Y314,S280 | Y473,H449  H323 | Y473,Y449  H323,S289 |
|  | -12.49 | -13.50 | -8.74 | Y464,H440  Y314,S280 | Y473,H449  H323 | Y473,Y449  H323 |
|  | -12.54 | -13.00 | -13.01 | Y464,H440  Y314,S280 | Y473,H449  H323,T288 | Y473,Y449  H323,S289 |
|  | -11.40 | -12.40 | -12.58 | Y464,Y314  S280 | Y473,H449  H323,T288 | Y473,Y449  H323,S289 |
|  | -12.40 | -13.40 | -12.39 | Y464,H440  Y314,S280 | Y473,H449  H323,T288 | Y473,Y449  H323,S289 |
|  | -12.00 | -13.51 | -13.49 | Y464,H440  Y314,A333 | Y473,H449  H323,T288 | Y473,Y449  H323,S289 |
|  | -10.95 | -11.56 | -11.88 | Y464,H440  Y314,S280 | Y473,H449  H323,T288 | Y473,Y449  H323,S289 |
|  | -11.64 | -10.93 | -12.99 | Y464,H440  Y314,S280 | Y473,H449  H323 | Y473,Y449  H323,S289 |
|  | -12.09 | -12.49 | -13.11 | Y464,H440  Y314,S280 | Y473,H449  H323 | Y473,Y449  H323,S289 |
